# Cognition Assessment Technologies on Deaf People

**DOI:** 10.5334/joc.262

**Published:** 2023-03-08

**Authors:** Coral I. Guerrero-Arenas, Fernando Uristy Osornio-García

**Affiliations:** 1 Departamento de Ingeniería en Sistemas Biomédicos, Universidad Nacional Autónoma de México, Ciudad de México, MX

**Keywords:** Deafness, executive functions, technology

## Abstract

In recent years there has been a growing interest in research about the different ways of processing and consolidating cognition in deaf people. It is known that hearing loss can lead to differences in some executive functions like control inhibitory or working memory. This literature review describes executive functions in deaf people and how they could be evaluated through technological devices complementing traditional assessments, like neuropsychological batteries. We identified biometric devices, digital and physical interfaces, and software from the literature, whose goal is to design or adapt technology to assess some cognition domains in several ways. The results of the review suggest the need to understand the cognitive phenomenon that significantly impacts the context of deaf people; moreover, it becomes relevant as a line of research in the Cognitive Science of Hearing. Using technologies to measure them and gain a better understanding of cognition in deaf people may provide possibilities for designing or adapting targeted educational or therapeutic strategies.

## Introduction

The executive function (EF) construct encompasses a set of higher-order cognitive abilities such as the organizational, control, and self-regulation skills necessary for goal direction (Cristofori, Cohen-Zimerman & Grafman, 2019; Figueras et al., 2008). It has been reported that people with hearing loss may have a higher risk factor for developing and consolidating some of these executive functions (Mason et al., 2021; Conway, Pisoni & Kronenberger, 2009; Pisoni & Cleary, 2003). No agreement yet on the consequences of the hearing loss itself or the social and personal restrictions are underlying the barrier to communication with others (Corina & Singleton, 2009) or in addition to other factors such as the etiology of hearing loss and sociocultural context.

Particularly some researchers have exposed differences in attention skills (Bavelier et al., 2000a; Hauser et al., 2007; Thakur et al., 2019; Tharpe et al., 2008; Zeni et al., 2020) in working memory tasks (Burkholder & Pisoni, 2003; Pisoni & Cleary, 2003), inhibitory control, (Mason et al., 2021), and sensorimotor synchronization (Petry et al., 2018; Tranchant et al., 2017). However, there is evidence for some auditory deprivation-dependent brain changes that may be related to functional and adaptive behaviors (Kral et al., 2016). Indeed, it has been hypothesized that hearing loss has effects beyond auditory processing and may affect cognitive functions related to sequentially and temporality since hearing is the primary sensory pathway for perceiving high-level sequential patterns that change in time rather than space (Conway et al., 2009). That is, that audition leads to the establishment of temporal ordering that underlies expectation and anticipation, what comes before and what happens next? For example, a subdomain of working memory attends to this temporality, as will be discussed below. It appears that deaf individuals, especially those who have not acquired language early, there may be more difficult in consolidating functions related to learning sequentially.

On the other hand, and in contrast to the above, it seems that once the sign language is consolidated there are increases in subdomains of some functions, for example, in peripheral attention processes, that underlie inhibitory control (Bosworth & Dobkins, 2002), this makes sense given that a visuospatial language such as sign language develops spatially, supported more by visual and proprioceptive processes. This is consistent with the Enhanced Hypothesis proposed by Rodger et al., (2021) and Sidera et al., (2017) which states that in the absence of hearing the visual system would have a more significant visual function. However, Dye, Hauser & Bavelier (2009) suggest that hearing deprivation is not a causal factor for presenting difficulties in cognitive processes, such as attention, but that these may be a consequence of other factors that have nothing to do with deafness *per se* since it is essential to take into account aspects such as the etiology of deafness, sociocultural factors, the age of acquisition of a communicative system, and even the modality in which this is consolidated, that is, whether it is a sign language or an oral language. Even the results that show differences in some cognitive domains are different in children and adults because the consolidation of neurodevelopmental milestones seems to depend largely on the acquisition of a communicative system, without conclusive evidence on the issue, mainly because of the dynamism that characterizes cognition and that must be considered in technological development.

Technological development has allowed converging lines of research related to the deaf population, emphasizing on time advance related to assistive technologies, especially those focused on communication. Assistive technologies are defined as those developments, software, or products that allow for to increase, maintain, or improve some functional capabilities in people with disabilities, for example, prosthetic pieces, hearing aids, screen readers, or wheelchairs, among others. Its main function is to assist the person to be as independent as possible. Most of the literature linking technology and deafness is focused on these types of devices, especially those developments to reduce communication challenges (Landolfi et al., 2022; Imran, et al., 2021; Jacob, et al, 2021; Albrecht, Jungnicke & von Jan, 2015).

For this paper we focus on technological advances that in recent years have made it possible to adapt devices and methodologies for different populations, allowing a better understanding of the cognitive processes mediated by hearing, but not since an assistance idea or to facilitate communication between deaf and hearing people. On the other hand, the design of technological tools where there is an outstanding commitment to the interaction with digital environments promotes better behavior and motivation in users, giving an advantage to the evaluation processes to be more dynamic compared to other traditional ones.

This review is divided into three parts. The first part shows a brief description of the executive and cognitive functions; in part two we will give an account of the use of technologies such as interfaces and biometric devices, and the development or adaptation of software for the assessment of these functions. Finally, we conclude with a summary and perspectives on future applications and considerations for developing or adapting technological devices that complement the assessment of executive functions in the deaf population.

We introduce a qualitative literature review to explore if there are technological devices that evaluate executive functions or at least one of them, to show alternatives or complement neuroimages techniques or neuropsychological batteries, especially for the difficulties explained before the communication barrier. For this review, we exclude assistance or teaching devices, because the goal was not to evaluate this competence or the design adaptation for physical or communication nor aid hearing. The cochlear implant variable or another hearing aid did also not include because is documented that the cognitive process is usually different in people with hearing aid (van Wieringen et al., 2019; Hua, et al., 2017).

### Search strategy and selection criteria

We searched PubMed and Web of Science, with terms like “deaf”, “hearing loss”, “hard hearing”, “technology devices”, “executive functions”, and “cognition assessment”. Terms excluded “aid hearing”, “cochlear implant”, “assistance device”, “therapy”, “education”, and “rehabilitation”, for reports published between January 2011 to November 2022. Our main focus was on studies that assessed one or more executive functions or cognitive skills through biomedical devices or using technology but not neuroimaging, in deaf people.

This was an example of a formula used in research:

*deaf people AND cognition AND technology NOT hearing aid NOT cochlear implant NOT education NOT therapy*

We do not include articles published in languages other than English, either type paper-like reviews, conferences, and not available in full text. We decided in this way because most of the research or interventions are focused on teaching sign language or another linguistic topic as well as restoration or hearing assistance.

## 1. Cognitive and executive functions

The construct of executive and cognitive functions has been coined from different perspectives over time. Lezak (1982) distinguished both based on what was intended to be known for each function. To describe cognitive functions, the author emphasized how much knowledge or skills the person possessed; for example, what skills remained intact or were impaired in people, or how well they performed a task compared to another, and on the other hand, executive functions are related to whether people do something or not, i.e., they focus on execution and what is related to it. Barkley (2012) contributes to this meaning by indicating that executive functioning underlies how a person achieves a goal efficiently. Lezak (1982) indicates that we should ask ourselves: ask how well the patient maintains a performance rate, how consistently and effectively he self-corrects, and how responsive to changes in the demands of the task, for example.

Recently, executive functions (EFs) have been described as a series of mental processes necessary to be attentive, solve problems, reason, have self-control, and persist in a task (Blankenship et al., 2019; Kral et al., 2016). According to some authors, EFs are framed in three central components, and, from them, other dimensions are derived that are supported by the first ones: working memory, inhibitory control, and cognitive flexibility (Diamond, 2020; Diamond, 2013), Cristofori, Cohen-Zimerman & Grafman, (2019) also include planning, reasoning and problem-solving. For this review, we consider the three central components and the subparts or dimensions associated with each of them, especially attention, which is framed within inhibitory control and seems to be the most studied and controversial function in deaf people.

Inhibitory control is required to control behaviors, thoughts, and emotions, and to adapt actions to emit or not a response. It is therefore strongly linked to cognitive control and working memory (Cristofori et al., 2019). This ability supports attention because it is necessary to inhibit external and internal factors and sustain attention to achieve specific goals. Attention is understood as the ability to filter, select, and focus on various elements of the environment (Dye, Hauser & Bavelier, 2008). To this effect, Hauser, Lukomski & Hillman (2008) indicate that inhibition is one of the major cognitive skills because it models motor and behavioral control. It has recently been theorized that inhibitory control is not a single unit, but is composed of a series of functions, among which two main ones are differentiated, response inhibition, which refers to the ability to control impulsive behavior to prevent (inhibit) motor and verbal responses, and interference suppression that involves working memory and refers to the ability to suppress interfering information (Daza González et al., 2021).

Working memory (WM) involves the ability to retain information in the short term, even when it is not perceptually present, as well as the ability to manipulate that information before it passes into long-term memory (Diamond, 2020; Baddeley, 2017). Two types of WM are generally distinguished, visuospatial and verbal. WM is critical for making sense of anything that unfolds over time, for that always requires holding in mind what happened earlier and relating that to what comes later (Diamond, 2013). The reasoning is closely linked to WM because you need to have the information online long enough to be able to manipulate it and act accordingly.

Lastly, cognitive flexibility is built on WM and inhibitory control and is defined as the ability to adapt in the face of environmental change and to generate new ideas that drive innovation and promote growth and discovery (Cristofori et al., 2019; Badre and Wagner, 2006). Cognitive flexibility is associated with an ability to adapt to changing conditions in the environment, which is why it requires control and manipulation of information to know new strategies.

Some researchers have been exposed to differences in EFs in deaf people, in particular in attention skills (Zeni et al., 2020; Thakur et al., 2019; Tharpe et al., 2008; Hauser et al., 2007; Bavelier et al., 2000b); the execution of tasks of working memory (Mason et al., 2021; Cardin et al., 2018; López-Crespo et al., 2012; Burkholder & Pisoni, 2003; Pisoni & Cleary, 2003), and inhibitory control (Mason et al., 2021). It seems to be that some executive skills are so close to the linguistic domain that are delayed consolidating (Figueras et al., 2008), maybe because deaf people acquire a late sign language, opposite to oral language that is developed since birth. Often, deaf children do not have access to a language at birth due to the majority being born in a hearing family or because the educational system does not give them an effective early intervention in sign language (Krebs et al., 2021). Thus, the sociocultural context also seems to affect the development and consolidation of some EFs, especially when a deaf child is born into a hearing family environment.

On the other hand, EFs seem to be linked with the fact that, when there is sensorial deprivation, the remaining senses appear to be more accurate, which is consistent with the Sensorial Compensation Hypothesis (Pavani & Bottari, 2012). Whereas where in the case of deafness, vision, and tactile sense are more sensitive to inputs from the environment. From the neurocognitive perspective, this is due to the brain’s ability to reorganize connectivity to other areas that are not affected by the sensory loss, in this case, there is a major recruitment of visual and tactile areas, although this process of neuroplasticity depends on variables such as the environment, age of onset of deafness and even interindividual factors that influence brain adaptation to sensory loss (Kral et al., 2016). The authors emphasize the possibility that, as the brain is a dynamic system that bases its development on experiences between neural activity and stimulation from the environment, a sensory loss could affect, positively or negatively, domains beyond hearing. This could be concordant with an enhancement in attention skills, for example. The case of disturbance in inhibitory control and working memory seems to be based on hearing feedback, which is coincident with *Auditory Scaffolding* proposed by (Conway et al., 2009) where some cognitive domains are underlying in sound perception, especially that based on temporally and sequential processing, like working memory.

## 2. Use of technology to assess cognitive domains in Deaf people

One of the most common methods to assess EFs is through neuropsychological tools. However, in the case of deaf people, there are two main factors to take into consideration: first, the communication barrier, since most of the batteries are designed for hearing people, and second, the consideration of the delay in language acquisition as a risk factor for the consolidation of cognitive functions. A systematic review by Vázquez Mosquera (2021) indicated that there are no rigorous instruments with special adaptations to assess deaf children, in this case. This could be a reason that some authors combine technology and assessment cognitive functions. We found design research for deaf children, named AWARD Neuropsychological battery, developed by González et al., (2011) that explores cognitive areas from tasks executed in a web application, where they can configure the language depending on user preference, for their research, could be *Lengua de Signos Española (LSE)* or oral language. This software tool allows making a cognitive profile of deaf children by implementing a battery of neuropsychological tests using adaptive web technology, whose main utility would be that professionals in neuropsychology can make use of this tool to minimize communication barriers. It should be noted that, although technological devices are accurate in detecting and evaluating cognitive functions, the design of tasks/paradigms should be more sensitive to detect changes in what is to be evaluated, considering that no brain function occurs in isolation, but rather the sum of interactions of various processes.

Based on the literature consulted for this review and taking into consideration that the exploration of EFs in deaf people is less explored compared to studies conducted on hearing people, the studies found so far in this review are described. The sections are divided according to the classification of assessment tools used by the authors of the studies. However, we know that each one had a different population of studies, in terms of adults and children, so we try to limit them to deaf people without cochlear implants, although we emphasize that we cannot generalize the results to the entire deaf population, since the heterogeneity of people must be considered. Within the literature review, we found a wide variety of technological devices that can be used to assess EFs, without most of them being necessarily designed for the deaf population, but rather have been adapted from traditional use in research in other populations and where the paradigms have been adapted by interpreting the instructions into some sign language, for example. That is the reason why the studies shown in this review include technological developments that can be classified as biometric devices, physical and digital interfaces, and video games. This review is an attempt to link the use of technological devices to assess these functions and to give a prospective scenario for future developments and applications. The papers reviewed are in Table 1.

**Table 1:** List of reviewed articles since 2011 to 2022.


YEAR	TYPE OF COGNITION	TYPE OF TECHNOLOGY	REFERENCE

2011	Visual attention	Eye tracker	(Watanabe et al., 2011)

2012	Working Memory	Software/interface	(López-Crespo et al., 2012)

2013	Visual perception	Software/interface	(Barca et al., 2013)

2014	Attention and cognitive control	Software/interface	(Dye & Hauser, 2014)

2015	Visual Attention	Eye tracker	(Heimler, van Zoest, Baruffaldi, Rinaldi, et al., 2015a)

2015	Visual Attention	Eye tracker	(Heimler, van Zoest, Baruffaldi, Donk, et al., 2015b)

2018	Visual Attention	Eye tracker	(Worster et al., 2018)

2018	ANS/Inhibition and Working Memory	Software/Interface	(Bull et al., 2018)

2019	Visual attention	Software/interface	(Thakur et al., 2019)

2020	Computational thinking	Software/interface/ vibrotactile device	(Cano et al., 2020)

2020	Visual attention	Eye tracker	(Krejtz et al., 2020)

2020	Visual attention	Eye tracker	(Zeni et al., 2020b)

2021	Emotional arousal and Workload cognition	Eye tracker	(Tsou et al., 2021)

2021	Visual attention	Eye tracker	(Bonmassar et al., 2021)

2021	Inhbitory control	Software/interface	(Daza González et al., 2021)

2022	Visual attention and inhibitory control	Software/interface	(Dye & Terhune-Cotter, 2022)


*Note*: The table shows the type of cognition evaluated in each research and the technological device used for them.

### 2.1 Biometric assessment: *Eye tracking*

According to the literature consulted for this review, eye tracking is one of the most widely used tools for the evaluation of processes related to visual function, particularly visual attention. The eye-tracker is a biometric sensor technology for recording eye movement and gaze location. Eye-tracking is the process of determining the point of gaze or the point where the user is looking for a particular visual stimulus (Lim, Mountstephens & Teo, 2020). The data obtained can reveal how participants interact with stimuli on a global or specific level (Carter & Luke, 2020). Gaze is the externally observable indicator of human visual attention that can assess cognitive function and (Zeni et al., 2020) performance (Skaramagkas et al., 2021; Krafka et al., 2016). One of the advantages of using eye-tracking is that, depending on the task presented, eye movements can be voluntarily controlled and evaluated for speed and accuracy. Its use within the cognitive area is because visual orientation relates the oculomotor system to cognitive and neurological processes (Carter and Luke, 2020), so accurate inferences can be made about observable behavior and mental state during the performance of visual tasks through estimates of eye and pupil behavior. There are eye-tracking devices with monocular technology that acquire information from only one eye. Theoretically, this should be sufficient since the movements of one eye mimic those of the other, i.e., the eyes tend to move together in the same direction, so knowing the position of one eye can predict the position of the other eye as well. However, binocular acquisition is usually used because this type of biometric data recording reduces errors in precision and accuracy since the on-screen position of both eyes is averaged. On the other hand, there are eye-tracking systems where infrared sources illuminate the eye while a series of cameras, also infrared, capture its reflection, calculating its relative position concerning the physical world. This is because infrared light is reflected in two parts of the eye, the retina, which helps to calculate the center of the pupil (pupil reflection), and the cornea (corneal reflection) (Brunyé et al., 2019). The corneal reflection usually does not change when the eyes move, while the pupil reflection does.

#### 2.1.1 Eye-tracking and cognitive functions

It appears that hearing loss may underlie visual abilities that are reflected in processes such as attention, which is defined as the general alert to interact with the environment (Lindsay, 2020); and there is the ability to focus perception on one or a group of stimuli by suppressing those that are irrelevant (Diamond, 2013). According to some authors, deaf people seem to use other visual resources or strategies to appropriate and link with the environment. For example, they tend to fixate gaze on or near the eyes and hearing people prefer to fixate gaze at or near the mouth (Heimler et al., 2015a). The development of visual attention and gaze tracking has been the subject of study in the neuropsychological field because they are closely related to attentional control and social cognition, as it accounts for how visual resources are redistributed to interact with and understand the surrounding world. This is clearly seen in the case of deaf infants of hearing parents, where there is a redistribution of attentional resources by having to focus on several stimuli at once, for example, while the caregivers point to something and name it. Deaf infants have to focus on the caregiver, then on what is pointed to, and then on the caregiver again to appropriate that external element. In the case of hearing infants, they only must see the object while the auditory input is present. This can be considered as a precursor to the consolidation of attentional control, and it refers to perception control to focus the mental resources on specific things, so the attention works at the same time as inhibitory control. Visual attention is understood as the process required to isolate relevant information from non-relevant information within a visual scene (Skaramagkas et al., 2021). Within this visual processing, two main attentional functions are evaluated, overt attention and covert attention. In the former, there is a selective orientation towards an object or spatial situation and the observable response is eye movement directed in some direction. Covert attention involves a change in the focus of attention, but no change in eye position or eye movement. Blair and Ristic (2019) indicate that this type of attention evidence how mental resources are aligned with the target of the response, whereas overt attention recruits oculomotor resources toward that target, manifesting in an observable response. Within these overt and covert attention processes is the research of Bonmassar et al. (2021) who observed that the interaction between both types of attention is different in people with deafness, in particular in spontaneous over-eye movement performance when there are social and non-social central cues. The authors observed that deaf participants were slower to respond compared to hearing participants. Bottari et al., (2012) complement this information by indicating there may be a neuroanatomical and functional reorganization underlying eye control when executing covert attention tasks. Also observed that voluntary and reflexive eye movements were more prominent in deaf compared to hearing people.

Zeni et al., (2020) used a context of complex naturalistic scenes, observing that deaf people prioritize objects within the images, which leads to infer that attentional differences may refer to an object and not only to the spatial or peripheral context, as previously described, which is consistent with the work of Heimler et al., (2015b) who observed that deaf people have greater control in gaze shifting when monitoring visual space when they are immersed in a social situation. This may be because they require a balance between maintaining spatial attention on the faces of their interlocutor to ensure better communicative and social quality and effective monitoring of the environment. The authors especially emphasize that this control may depend on the experience of acquiring sign language, which makes sense given that it is framed as a visuospatial system that unfolds in a signed space. Given the above, it is important to highlight the design of paradigms for the study of attentional processes. The aforementioned studies used complex contexts which although they are susceptible to involve other cognitive resources, is how it occurs in everyday situations. Another function of attention concerns the allocation of visual resources, which is measured within visual parameters with fixation metrics and saccades. Perhaps one of the goals of recording eye movements can provide information about recognition processes in social situations. For the study of cognition in deaf people, this is of prime importance, because from the knowledge of these parameters it is possible to know the differences between social dynamics when recognizing facial expressions. For example, for the recognition of emotions, there is a greater prominence of visual allocation in the eyes and mouth. Krejtz et al., (2020) examined visual attention while encoding emotions from facial expressions. They found that there was greater accuracy in detecting expressions related to happiness, although there were no significant differences between deaf and hearing. What they did find was a tendency to be faster at recognizing emotional expressions, which they hypothesize may be related to a more prominent reactivity to movement within a peripheral visual field. It was also observed that deaf people directed less attention to the mouth area and more to the eyes. Even a dynamic gaze pattern from ambient to focal was observed, especially during the detection of happiness-related stimuli. In line with these authors, Watanabe et al., (2011) observed that deaf participants also had higher fixation times in the eye and nose area in emotion recognition tasks, confirming that eye contact is a crucial component in processes related to emotions, social skills, and world appropriation.

It is interesting to take up again the differences in the allocation of attention between adults and children. As mentioned, deaf adults seem to spend more time looking at their interlocutors, while children interpret social situations by allocating their visual resources, first and for a shorter time, to the person’s face and then to the person’s body, i.e., they divide their attention to process emotions more effectively within a social context (Tsou et al., 2021). This is consistent with the arguments of Corina & Singleton, (2009) who state that deaf children of hearing parents have a disadvantage in consolidating their attentional process, because not having auditory input, they necessarily must divide their attention between their caregivers and the outside world, i.e., the perceptual and cognitive challenge is greater compared to a hearing child, as mentioned above. However, Worster et al., (2018) observed no differences between deaf and hearing children in speech-reading tasks, so they assume that hearing loss would not necessarily affect gaze behavior. In agreement with the studies of Heimler, et al., (2015a) and Watanabe et al., (2011), Worster et al., (2018) observed that gaze patterns were like those of adults, alternating between eye-mouth-eye gaze. This reinforces the argument that, for deaf people, visual expression contains more socially relevant information compared to other facial elements.

As can be observed, the use of biometric tools such as eye trackers to measure functions like visual attention seems to be the most used technology, and perhaps under the assumption that deaf people use other visual mechanisms which may be mediated by several factors such as the acquisition of a sign language, a neuroplasticity effect due to sensory deprivation and even the sociocultural context in which they develop. However, a limitation that arises is the possibility that, as people know that their eyes are being monitored, this may lead to a different behavior in eye tracking, given that the use of eye trackers may affect, in an unconscious way, the movement of the eyes, which would lead to other types of information (Bonmassar, et al., 2021). On the other hand, mapping these visual strategies from eye-tracking allows us to elucidate the visual function underlying other functions, e.g., social skills or another related to reading and writing.

### 2.2 Digital and Physical Interfaces

For this review we found other types of developments that are usually used in research, and that have been adapted for other populations. In this section, we include the interface can be defined as any medium, whether physical or virtual, that allows the interaction between a subject and a machine (Pérez-Ariza & Santís-Chaves, 2016). The area that specialized in the development of interfaces is called Interaction Design. Norman (2013) defines it as the branch of design that focuses on understanding the nature of the interaction between people and technologies. The development of technological tools has provided multiple possibilities to evaluate the interactions and behaviors of individuals in front of interfaces to improve them, causing an enhancement in the user experience. For this, software and hardware designs must comply with the usability components, both in the functional development of the system itself and in how people interact with it.

Within the classification of interfaces are haptic interfaces, described as devices that allow interaction/communication between person and machine through touch, which may or may not be accompanied by auditory or visual stimuli. Their main characteristic is that they are activated in response to the user’s motor action (Hayward et al., 2004). Haptic interfaces are categorized as mechatronic devices because they take advantage of mechanical signals for this exchange of communication between people and machines (Hayward et al., 2004). Among the simplest haptic interfaces are keyboards, buttons, and others more sophisticated such as gloves and exoskeletons.

The following are elaborated from a categorization of the digital and physical interfaces found for this review. We describe the use of software and interfaces adapted for the assessment of the specific domains of attention and working memory, followed by using games as an assessment tool.

#### 2.2.1 Digital and Physical Interfaces and EFs

Dye and Hauser (2009) used the Gordon Diagnostic System, a portable microprocessor-based continuous performance test that assesses inattention and impulsivity-based behaviors (Foster, 2011). Although the GDS was initially developed to assess children and adolescents with ADHD, the literature reports that children with hearing loss have difficulties maintaining attention and cognitive control (Mason et al., 2021). One of the characteristics of the GDS is that it evaluates through Continuous Performance Tasks (CPT), defined as streams of stimuli that change continuously, and where randomized objective stimuli appear. Participants then must respond to this specific stimulus (Roebuck et al., 2016). Dye and Hauser (2009) evaluated sustained attention, selective attention, and cognitive control, and found that deaf children have a lower performance in the execution of attention and cognitive control tasks, perhaps contingent on the late acquisition of a sign language, so they suggest extending their research to deaf children of deaf parents because in this way it is inferred that they are exposed to a natural language from birth, since in a Deaf family a sign language is the natural language for communication, in the same way that a hearing family communicates orally. A relevant finding in this study was that the participating children were profoundly deaf and had access to a sign language since their deaf parents, and in the results no significant differences were observed between hearing and deaf children, suggesting that hearing deprivation is not sufficient to find significant differences in reported outcomes on attention, as mentioned above. Dye and Terhune-Cotter (2022) conducted a 3-year cohort of deaf children to determine whether there were changes over time in sustained selective attention processes or inhibitory responding and whether these changes were influenced by language and/or deafness. On this occasion, the authors found a significant correlation, observing that learning English is more closely related to better selective attention processing, while the acquisition of American Sign Language (ASL) is more closely related to better inhibitory control. They speculate that the above is due to two factors: the temporal precedes the acquisition process. It is emphasized that ASL is learned through natural exposure to meaningful communicative interactions, i.e., it is an acquisition process. While for English this does not occur, it is learned through therapies and generally after critical periods of neurodevelopment. This seems to be associated with the fact that acquisition occurs spontaneously and unconsciously within a natural context of language use (Krashen, 1982), and on the other hand, the process of learning a language is characterized by a conscious knowledge of rules and their use, which is not reflected in a fluent production of it.

Dye and Terhune-Cotter conclude that there is a close relationship between language and cognitive skills, especially in selective attention and inhibitory responding. In this research, the authors indicate that they used a version of the Gordon Diagnostic, the vigilance form, and recommend that they use other types of experimental approaches, which agrees with the findings of the study by Foster (2011) it also recommended that to validate the results obtained from the GDS, it should be combined with other assessment resources.

One of the functions that seem to be most compromised in deaf children is working memory (WM). As mentioned, it is a type of short-term memory whose goal is to be able to retain and manipulate information for a short time before it disappears, and which, according to Baddeley’s Working Memory Model, is determined by two subsystems based on sensory modalities: 1) the phonological loop, underlying the auditory system, and 2) the visuospatial sketchpad, modulated by the visual and spatial system. It seems to be expected that deaf people have better strategies to perform visuospatial tasks, based on two main assumptions: 1) the enhanced hypothesis or sensory compensation hypothesis, which explains that in people where there is a loss of some sense, other sensory modalities are increased to compensate for the one that is compromised, in the case of deaf people, it would be expected that the visual system incorporates greater resources that would allow greater interaction with the environment, perhaps because the acquisition and use of a visuospatial gestural language that develops within a sign space.

López-Crespo et al., (2012) used a computerized version of a DMTS task for visual WM assessment to estimate whether different modes of communication (sign language, oral, or both) determined whether the resources to execute visual WM tasks were different compared to hearing children. Contrary to expectations, deaf children performed less than hearing children, even when the tasks were spatially based. This does not imply, as mentioned by the authors, that there is a delay in WM but perhaps there is another type of functional processing. This study makes it relevant to establish within the methodology the communication mode as one of the variables to have reliable results, i.e. sign language, oral, or both. Recently, Daza González et al., (2021) assessed inhibitory control ability using a computerized Stroop task and a short version of the Attention Network Test for children. This research aimed to observe whether the frequency of inattention, impulsivity, and hyperactivity behaviors, as well as disruptive, aggressive, and antisocial behaviors, was higher in a deaf group than in a matched hearing group. And the second aim was to determine whether any behavioral differences between deaf and hearing children could be explained by deficits in inhibitory control. This is due to the idea that deaf children present greater behaviors related to inattention and impulsivity and that this can represent cognitive and emotional difficulties; however, Daza Gonzalez’s research showed no significant differences between deaf and hearing children in terms of inhibitory control tasks. The conclusion reached by the authors is that the behaviors presented by deaf children are adaptive visual coding strategies to obtain information from their environment. These visual strategies require redistributing their attentional resources to their central visual field but also to the periphery, which requires a shift of attention to the environment more frequently than hearing people, which may be perceived by others as inattention or hyperactivity (Daza González, et al, 2021).

From the manipulation of information in a short period and from the apprehension of abstract concepts other functions are also consolidated, for example, the approximate number system (ANS), which refers to a cognitive process where estimates of magnitude are made from abstract representations. This requires other general domains, such as working memory and inhibition, for example, Cai et al., (2018) and Bull et al., (2018) assessed ANS using Superlab 4.0 software and a button-press interaction interface. In the end, it was observed that a deficit in general domains leads to lower performance in ANS task execution, which was more evident in deaf children compared to hearing children, perhaps one of the reasons for this result is that ANS requires several skills such as WM, which involves retention of spatial and temporal information, as well as the allocation of selective visual resources and inhibitory control. The authors indicate that having a lower mastery of these skills impacts lower performance in ANS.

As can be seen, devices based on simple physical interfaces, such as a button or a keyboard, are the most common for evaluating tasks in children, maybe because the interaction with the device is so simple. The development of technological tools has provided multiple possibilities to evaluate the interactions and behaviors of individuals in front of interfaces to improve them, causing an enhancement in the user experience. For this, software and hardware designs must comply with the usability components, both in the functional development of the system itself and in how people interact with it. Usability measures the ease of interaction between the user and the interface (Chanchí, 2019) and is based on a set of attributes described by Nielsen (2013): useful, learnable, memorable, effective, efficient, and desirable for the user. In this sense, one growing field in research that meets the above criteria is games, described in the following section.

### 2.3 Games as tool assessment (Gamification)

Another way to evaluate is through the incorporation of games, especially when the population is children. One of the terms in trend is gamification, which refers to the use of games within a ludic context in which participants engage with the cognitive tasks presented, due to the combination of intrinsic and extrinsic motivation; and by integrating challenges and positive feedback, the need for competition is satisfied (Khaleghi et al., 2021). To interact with the environment, processing is required where multisensory interactions are shaped at neural, behavioral, and perceptual levels (Murray et al., 2016). Technology-based games can be used as a vehicle to assess cognitive skills or competencies involved in the game, but not to assess the game (Bellotti et al., 2013). The same authors indicate another category is the so-called serious games focused on being educational, but at the same time, fun/attractive. A review made by the authors mentions that the use of computational games is effective in some therapeutic fields and is related to the development of emotional, cognitive, and perceptual skills. A term that integrates these skills is Computational Thinking, which includes cognitive skills based on a constructionist approach, i.e. users build their knowledge from the understanding of their context (Cano et al., 2020). The use of digital strategies provides the opportunity to interact in different contexts, addressing and building new possibilities of mental processes (Ferreira, et al., 2021) that go hand in hand with a series of steps that give certainty to solving problems.

A game designed and developed for deaf children was based on a methodology of the same authors, called MECONESIS (Acronym in Spanish, MEtodología para CONcepción de juEgos Serios para nIños con discapacidad auditiva) (Cano et al., 2020). The game simulates a real environment with interfaces and physical elements simultaneously. They also made use of a vibrotactile and visual board, both generated simultaneously. The digital interface was a mobile application, where the children interacted through a QR code and where were instructed on the steps to follow. According to the authors, one of the challenges for the child’s development and engagement with the game was the interaction between the physical and digital devices. The authors’ work falls into two of the categories we propose for this review. One is the use of digital interfaces, and the other is the vibrotactile board that gives feedback to users as part of the developed game.

The combined use of games with other types of devices can be a viable option for the design of cognitive function assessment prototypes, considering the advantages that this offers. Khaleghi et al., (2021) even offer a framework to guide the gamification design process for the assessment of cognitive tasks. This can be a first approach for future research in the Deaf population, where the steps they propose are contemplated and with the background that has already been presented in this review.

## 3. Discussion

Interest in research on cognition in deaf people has been increasing in recent years, not only from the perspective of assistive, recognition, or educational devices but also for research and educational purposes. The evaluation of cognitive processes in deaf people allows inferences to be made from different perspectives:

From cognition. From the evaluation of different domains, it is possible to have deep knowledge about the differences in cognitive processes derived from hearing, which would be framed within the Cognitive Hearing Science proposed by Arlinger et al., (2009) that is aligned with an interdisciplinary approach between cognition and hearing. Maybe, although it may seem contradictory, from this field it is possible to rethink and rephrase approaches and research aimed at deaf people.

One of the limitations present in the reviewed articles is the heterogeneity of the population even between children and adults. The different etiologies and thresholds of hearing loss do not allow generalizations of the results to establish precise approximations about the development of executive functions. Although deafness is a risk factor for the development and consolidation of these functions, there is no consensus on whether deafness *per se* affects cognitive functioning or whether it is derived from the socio-cultural context, influenced by a lower interaction with the environment due to communication barriers; it has even been reported that deaf people have lower prosocial behaviors compared to their hearing peers.

This is also mentioned by Hauser, Lukomski & Hillman (2008), who argue that in most studies with deaf people, there is no care to select participants and that they are truly representative of the deaf community, so variables such as the age of acquisition of a sign language, socio-cultural context or etiology of deafness should be considered.

The evidence shows that there are differences between deaf and hearing people and that these differences do not imply disadvantages or negative issues for cognition in deaf people but seem to exhibit other types of strategies for the appropriation of the world, such as attentional or visual resources, like Daza González et al., (2021) proposed in their research. In this sense, we agree with the reflection of Marschark & Hauser (2008) that there is a misconception about the cognition of deaf people and what it is to be Deaf, which leads to a series of barriers in different areas such as work, school, social and even family.

One of the EFs that seems to have the greatest difficulty in consolidation is working memory, especially in deaf children. Understanding that differences in this function may underlie learning in mathematics, reading, coordination in motor skills, abstract concept formation, and categorization should be basic to better academic achievement, for example. Auditory experience provides temporal patterns that help develop the above skills, so it would be expected that Deaf children would show difficulties in consolidating areas related to sequential processing, but this is not general for all people. On the other hand, several authors describe advantages in visual processes, particularly those referred to as peripheral visual attention, with emphasis on people who have already acquired sign language, perhaps because visuospatial languages develop in space. However, this visual advantage may entail difficulty in deaf children, because, not having developed inhibitory control, it is common for them to be labeled as distractible or inattentive, although the cognitive basis indicates otherwise. It is imperative to note that one of the variables that stands out as a differentiator in the development of EFs is the early acquisition of sign language. In different contexts, it is known that children are the first generation of deaf people in their hearing family, which hinders the acquisition of a mother tongue in the first years of life, and it is not until a later age that children can have access to sign language. It is worth noting that at this point we would no longer speak strictly of language acquisition, which would occur spontaneously in a natural context (Krashen, 1982), but of language learning, where there is an express search for the linguistic forms to be used and which will allow access to communication resources (Muntzel, 1995).

Understanding the basis of the cognitive dimensions in the deaf population allows them to rethink conceptions about their abilities, to design educational strategies that enhance the advantages and help those domains with greater difficulty, and although it is emphasized that neither the results nor the inferences are generalizable to the entire deaf population, it can be a starting point for lines of research and action.

From technology. The use of digital devices seems to be increasing for the evaluation of different cognitive processes. In this sense, and within the biometric data devices, the use of eye trackers seems to be the most usual, based on the premise that deaf people have different visual strategies than hearing people, due to the expanded use of space by the form of visuospatial communication. This, however, seems to be mediated by the consolidation of a communicative system, so in people who have not consolidated one, there seem to be differences in the results. This should be considered in the methodological conception to obtain the greatest homogeneity even when there is variability in the participants. One of the advantages of using technological devices to assess EFs is that they can provide more precise information about domains that may not be so evident in a traditional observation. This does not imply that assessment methods such as neuropsychological batteries should be replaced, but perhaps they can be complemented with other types of data that provide additional information for a better understanding of cognition. On the other hand, several authors refer to the use of a single sensory modality for the design of paradigms, so that integrating other sensory resources can provide more information about the processing of different cognitive functions. The current use of gamification seems to open possibilities of conjunction between learning and evaluation through a sense of playfulness, which brings other challenges in usability. As a future scope, the next revision could integrate virtual reality applications.

The development of technologies has been increasing and has diversified towards different needs of the deaf population. On the one hand, as teaching tools, to eliminate communication barriers by designing recognition devices or interfaces; others as assistive technologies based on electronic devices such as hearing aids or amplifiers, and the ones we focus on in this review, designs focused on the assessment of cognitive skills.

## Conclusion

The diversity of the use and adaptations of technologies shows the need to implement this type of device as a means, either complementary or primary, for the evaluation of skills and functions related to cognition and that move away from traditional methodologies. Perhaps a line of research that can provide accurate approximations of the development of executive functions is focused on the study and evaluation of different cognitive strategies and tools that would be mediated by the absence of auditory input. In this sense, assessment through technology offers the opportunity to observe and make estimates about cognitive domains and subdomains in deaf people. Although there is ample evidence of the use of standardized systems, such as the eye tracker, or some interfaces, such as the Gordon System Diagnostic, we identified that there are other devices that have not been validated in large populations, which is a limitation mentioned by the authors themselves. Another observation is that there is evidence that combining devices with other types of technologies, can yield more information on how and in what intensity the functions being evaluated occur (Pop-Jordanova & Pop-Jordanov, 2020). Approaches that integrate these parameters can be used to obtain more accurate, sensitive, and specific cognitive level indices. The development of technologies must then consider the interindividual, etiologies, heterogeneity, and sociocultural contexts in which Deaf people develop, and as a prospective scenario based on the above, consider interdisciplinary and collaborative work with the Deaf community itself to provide real and functional results that converge technological innovation and Cognitive Hearing Science.
